# Angiotensin II Overstimulation Leads to an Increased Susceptibility to Dilated Cardiomyopathy and Higher Mortality in Female Mice

**DOI:** 10.1038/s41598-018-19436-5

**Published:** 2018-01-17

**Authors:** Sophie Mathieu, Nabil El Khoury, Katy Rivard, Pierre Paradis, Mona Nemer, Céline Fiset

**Affiliations:** 1 Research Center, Montreal Heart Institute, 5000 Bélanger, Montréal, Québec, Canada; 2 Faculty of Pharmacy, Université de Montréal, Montréal, Québec, Canada; 3 Department of Pharmacology and Physiology, Faculty of Medicine, Université de Montréal, Montréal, Québec, Canada; 4 Lady Davis Institute, McGill University, Montreal, Québec, Canada; 5 Ottawa University, Ottawa, Ontario, Canada

## Abstract

Heart failure (HF) is associated with high mortality and affects men and women differently. The underlying mechanisms for these sex-related differences remain largely unexplored. Accordingly, using mice with cardiac-specific overexpression of the angiotensin II (ANGII) type 1 receptor (AT1R), we explored male-female differences in the manifestations of hypertrophy and HF. AT1R mice of both sexes feature electrical and Ca^2+^ handling alterations, systolic dysfunction, hypertrophy and develop HF. However, females had much higher mortality (21.0%) rate than males (5.5%). In females, AT1R stimulation leads to more pronounced eccentric hypertrophy (larger increase in LV mass/body weight ratio [+31%], in cell length [+27%], in LV internal end-diastolic [LVIDd, +34%] and systolic [LVIDs, +67%] diameter) and dilation (larger decrease in LV posterior wall thickness, +17%) than males. In addition, in female AT1R mice the cytosolic Ca^2+^ extrusion mechanisms were more severely compromised and were associated with a specific increased in Ca^2+^ sparks (by 187%) and evidence of SR Ca^2+^ leak. Altogether, these results suggest that female AT1R mice have more severe eccentric hypertrophy, dysfunction and compromised Ca^2+^ dynamics. These findings indicate that females are more susceptible to the adverse effects of AT1R stimulation than males favouring the development of HF and increased mortality.

## Introduction

There are important sex differences in the incidence and aetiology of numerous cardiovascular diseases (CVD). Hormonal regulation may play an important role in altering cardiovascular function. It is well recognized that pre-menopausal women have a lower incidence of CVD than men of similar age and this relative protection is lost after menopause, suggesting that ovarian hormones and, in particular, estrogens, are cardioprotective^1^. Although the beneficial effects of female sex hormones on CVD are supported by experimental and observational studies^2,3^, there is still some controversy on this in light of the randomized clinical trial, Women’s Health Initiative (WHI) that failed to show cardioprotection of estrogen-progestin replacement^4^. Studies have shown that several functional sex steroid receptors are expressed in human and animal hearts^5–8^, and together these observations support a direct role for sex hormones in regulating cardiac function and expression of various targets during health and disease. Among the principal causes of death related to CVD, heart failure (HF) contributes to an important overall mortality rate and is associated with more than 50% mortality rate within the first 5 years following diagnosis^9^. Interestingly, sex hormones appear to be implicated in HF as there are important sex differences in the manifestation and risk factors associated with HF. Indeed, more women develop HF with preserved ejection fraction (HFpEF) whereas men usually suffer from HF with a reduced ejection fraction (HFrEF)^10–12^. Women are more at risk to develop HF if they suffer from hypertension or diabetes and have a lower survival rate post-MI^10,13,14^. Furthermore, Gerdts *et al*. have shown that before antihypertensive treatment, women had higher prevalence of left ventricular hypertrophy than men suggesting that they may be more susceptible to cardiac remodelling^15^. Left ventricular hypertrophy is an important risk factor of cardiac mortality and sudden cardiac death (SCD) and it remains unknown why women are more prone to develop a more severe hypertrophic phenotype^16,17^. According to the American Heart Association, in 2008 HF was responsible for the deaths of 124,598 men *vs*. 156,839 women^18^. Furthermore, hospitalization in the US for HF between 1980 and 2006 was higher for women than men, suggesting that women show a worse prognosis^19^. The reasons underlying this worse HF prognosis and higher sensitivity to the effects of hypertension remain largely unexplored and there is a need to understand the cellular and molecular underpinnings of these sex differences in order to treat women more appropriately and develop more effective therapies.

It has been shown that angiotensin II (ANGII), the main effector of the renin-angiotensin system (RAS), is a major mediator of hypertension and HF^20^. Indeed, the development of HF is associated with a chronic activation of the RAS where chronic ANGII results in structural and electrical remodelling of the heart^20^. Using transgenic mice overexpressing the human ANGII type 1 receptors (AT1R) specifically in cardiomyocytes, we previously demonstrated in a series of studies that chronic AT1R stimulation induces cardiac systolic dysfunction, hypertrophy and electrical remodelling in the absence of hypertension^21–24^. Nevertheless, it remains incompletely understood how various processes implicated in the development of HF, such as cardiac dysfunction, hypertrophy and ion channel remodelling differ between males and females as a result of chronic ANGII stimulation. Since women are more susceptible to develop HF in response to hypertension compared to men^10,14^, we hypothesized that females are more sensitive to ANGII which would contribute to the worsening of the pathology compared to males. Thus, in this study, we sought to address these questions by studying the direct impact of cardiomyocyte-specific AT1R overexpression on survival rate, cardiac systolic dysfunction, hypertrophy and electrical remodelling in both male and female mice.

Here, we report that the female AT1R exhibit a higher mortality rate compared to their male counterparts. This was associated with greater ventricular eccentric hypertrophy and dilation, an increase in cellular Ca^2+^ sparks frequency, smaller Ca^2+^ transients and slower Ca^2+^ reuptake in AT1R females compared to AT1R males. Together, these findings may contribute to the higher mortality rate in females with AT1R overexpression indicating higher sensitivity to ANGII and AT1R stimulation.

## Results

### Early and higher mortality rate is observed in female AT1R

**Figure 1:**
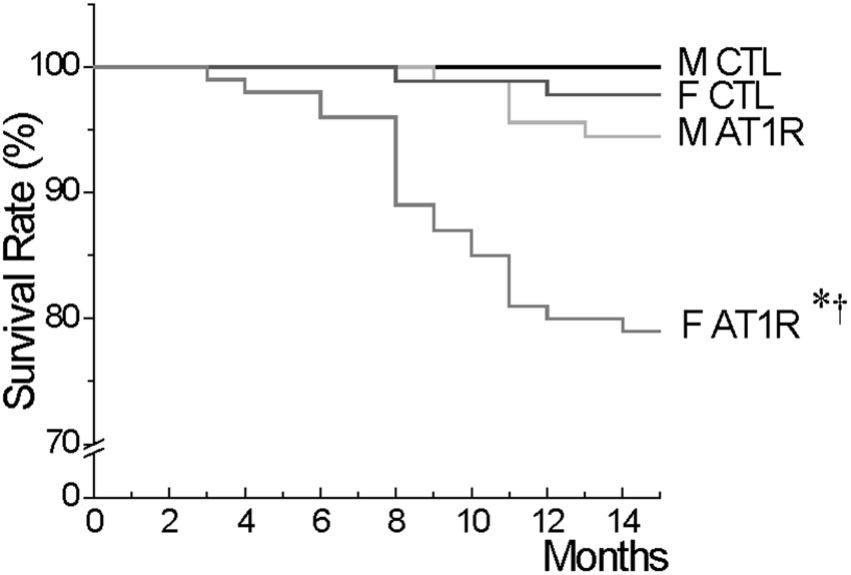
Survival curves reveals an early onset and higher rate of mortality in female AT1R mice compared to males. The graph represents the percentage of survival of CTL and AT1R mice of both sexes (M, F) between 0 and 15 months of age (^*^p = 0.0013 *vs*. F CTL, ^†^p = 0.0036 *vs*. M AT1R, Cox regression analysis) (M: CTL N = 107, AT1R N = 91, F: CTL N = 91, AT1R N = 100).

During the first 15 months of life, all deaths from the AT1R colony were collected and reported into a Kaplan-Meyer survival curve (Fig. 1). During the monitored period, the survival rate of female AT1R mice decreased by 21% *vs*. 5.5% in AT1R males compared to sex- and age-matched CTL. Importantly, the data also showed that female AT1R mice began to die around the age of 3 months whereas male AT1R mortality did not begin before the age of 9 months. We first ruled out the possibility that the higher mortality rate in female AT1R mice would be explained by differences in transgene overexpression or difference in circulating ANGII levels. Therefore, the *AGTR1* mRNA levels were determined and shown to be similarly increased (~700-fold) in the ventricles of male and female AT1R mice as reported in data supplement (Supplementary Table 1). Moreover, plasma ANGII levels were also comparable between control and AT1R mice of both sexes as also reported in Supplementary Table 1. Thus, these observations suggest that females are more affected by cardiomyocyte-specific AT1R overexpression and may present more severe cardiac alterations promoting their premature death.

### Ventricular electrical remodelling is similar between male and female AT1R mice

**Figure 2:**
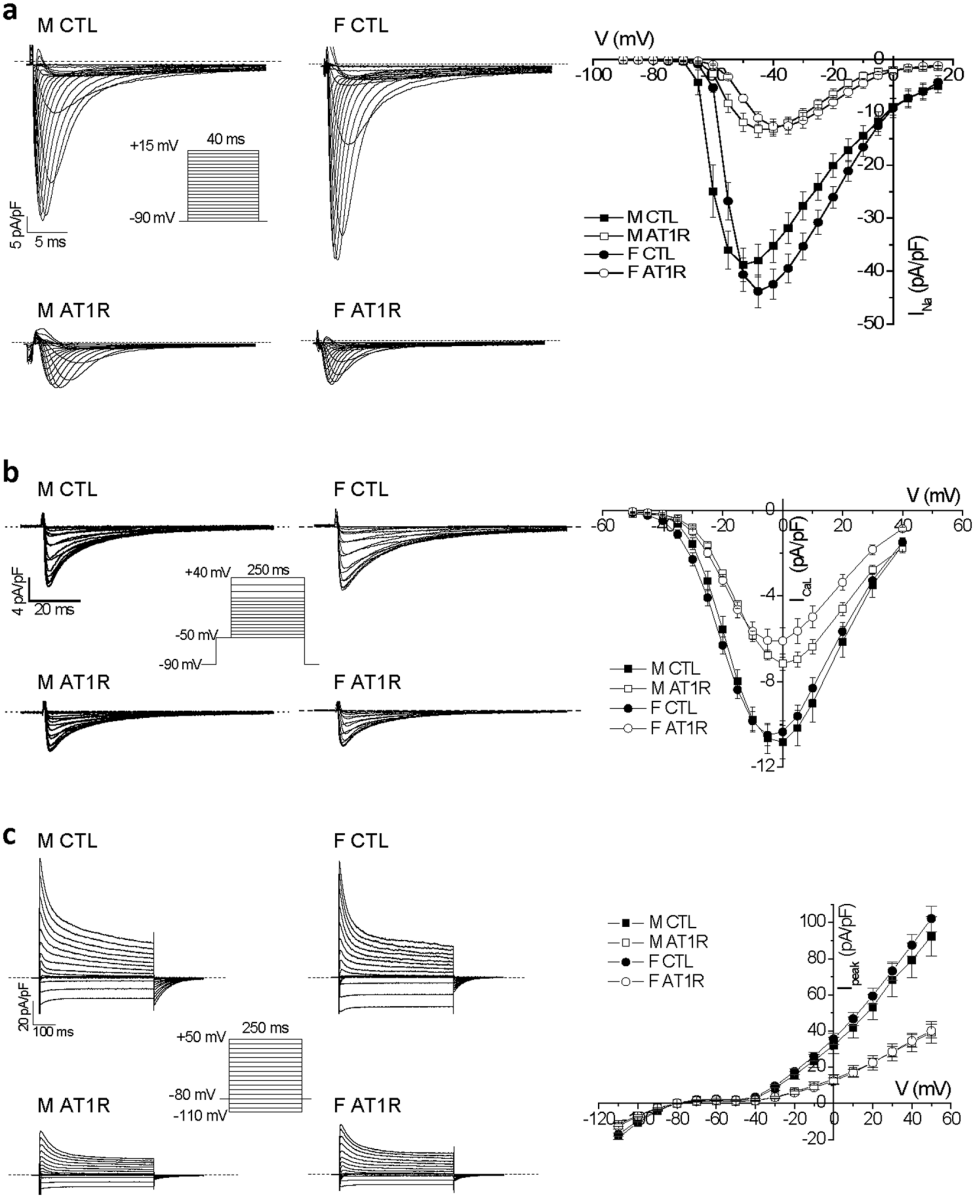
Ventricular myocyte ionic currents are similarly reduced in male and female AT1R mice in comparison to CTL. **(a**) Representative ventricular I_Na_ recorded in CTL and AT1R myocytes of both sexes. Mean data were plotted in a current-voltage (IV) relationships graph demonstrating that the reduction caused by AT1R overexpression is similar in male (M) (CTL: n = 13, AT1R: n = 16) and female (F) (CTL: n = 21, AT1R n = 18). (**b**) Typical I_CaL_ traces are presented and mean IV curves show that I_CaL_ is reduced to the same extent in male and female AT1R mice (M: n = 8, F: n = 10) in comparison to CTL (M: n = 6, F: n = 21). (**c**) Representative I_peak_ recordings from all groups are displayed. Mean IV curves also demonstrate that AT1R overexpression similarly reduces total K^+^ current (I_peak_) in males (CTL: n = 15, AT1R n = 11) and females (CTL: n = 13, AT1R n = 15). Voltage protocols used for the 3 types of ionic current are shown in insets. Male data for I_Na_ and I_peak_ presented in this figure represent a subset of the data presented in^22,24^.

We then examined ventricular electrophysiological properties between male and female AT1R mice in order to determine whether the higher mortality in females could be attributable to alterations in electrical remodelling associated with an increased risk of ventricular arrhythmias. We previously showed that the QRS complex, a measure of ventricular depolarization, was significantly prolonged in male AT1R mice^22,24^. Here we found that the prolongation of the QRS complex in female AT1R mice (CTL: 16.0 ± 0.8 ms, N = 9; AT1R: 21.2 ± 1.9 ms*, N = 6; *p < 0.05) was comparable to the data previously reported in males (CTL: 17.0 ± 0.6 ms, AT1R: 21.7 ± 0.6 ms*)^24^. Furthermore, ventricular repolarization assessed by measuring corrected QT (QTc) interval was also prolonged in female AT1R mice (CTL: 43.0 ± 1.0 ms, N = 9; AT1R: 49.7 ± 0.7 ms*, N = 5; *p < 0.05), consistent with values reported for males in a previous study (CTL: 44.5 ± 1.0 ms, AT1R: 49.2 ± 0.9 ms*)^22^. In line with these results, the density of the different ventricular ionic currents: the sodium current (I_Na_), the L-type Ca^2+^ current (I_CaL_) and the total K^+^ current (I_peak_) were all similarly reduced in AT1R male and female ventricular myocytes (Fig. 2). Overall, these electrical modifications, albeit severe, are comparable between the sexes and cannot account on their own for the higher mortality in female AT1R mice.

### Female AT1R mice suffer from more severe ventricular eccentric hypertrophy

**Figure 3:**
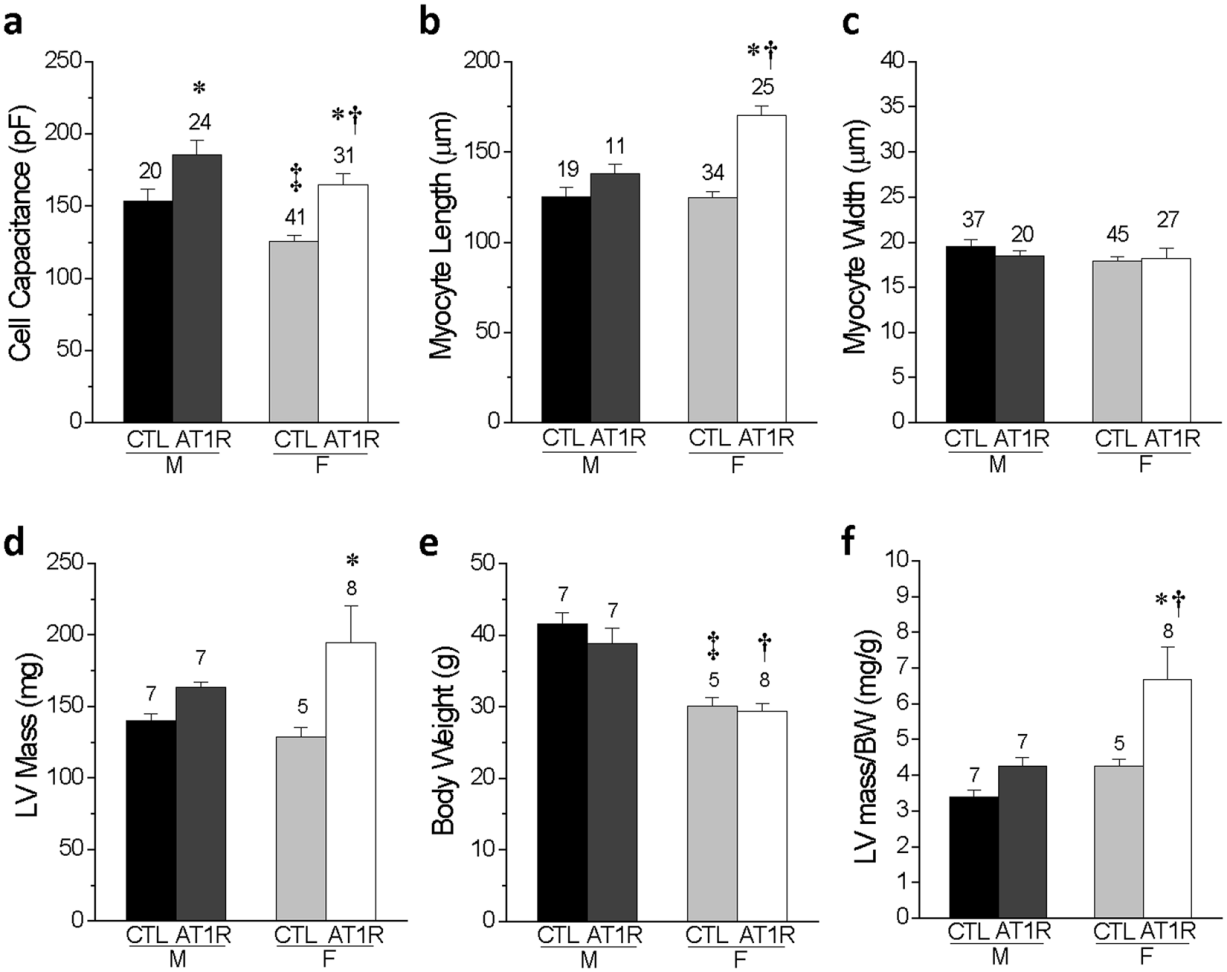
Female AT1R mice display more severe signs of cellular and ventricular hypertrophy in comparison to male AT1R mice. (**a**) Comparison of the cell capacitance measured in CTL and AT1R ventricular myocytes from male and female mice. (**b**,**c**) Comparison of the length (**b**) and width (**c**) of the ventricular myocytes isolated from CTL and AT1R mice of both sexes. (**d**) M-mode echocardiography was used to calculate the left ventricular (LV) mass for CTL and AT1R mice of both sexes. (**e**) Body weight (BW) and (**f**) LV mass/BW ratio is reported for each groups (*p < 0.05 *vs*. sex-matched CTL, ^†^p < 0.05 *vs*. M AT1R, ^‡^p < 0.05 *vs*. M CTL) (numbers above the bar graphs represent the number of cells or mice examined).

Since no differences in ventricular electrical properties were noted, we then evaluated critical components of HF related to systolic function and morphologic remodelling in these mice. At the cellular level, we found that cell capacitance was bigger in both AT1R groups and that ventricular myocytes were significantly longer in females AT1R in comparison to their sex-matched CTL (p = 2 × 10^−11^) and to the AT1R male (p = 0.0002) myocytes, without any differences in cell width (Fig. 3a–c). Data obtained by echocardiography from AT1R mice of both sexes showed that the left ventricular (LV) mass to body weight (BW) ratio was increased in female AT1R mice in comparison to AT1R male (p = 0.024) and female (p = 0.045) CTL (Fig. 3f) indicating a more pronounced hypertrophy of the female hearts.

**Figure 4:**
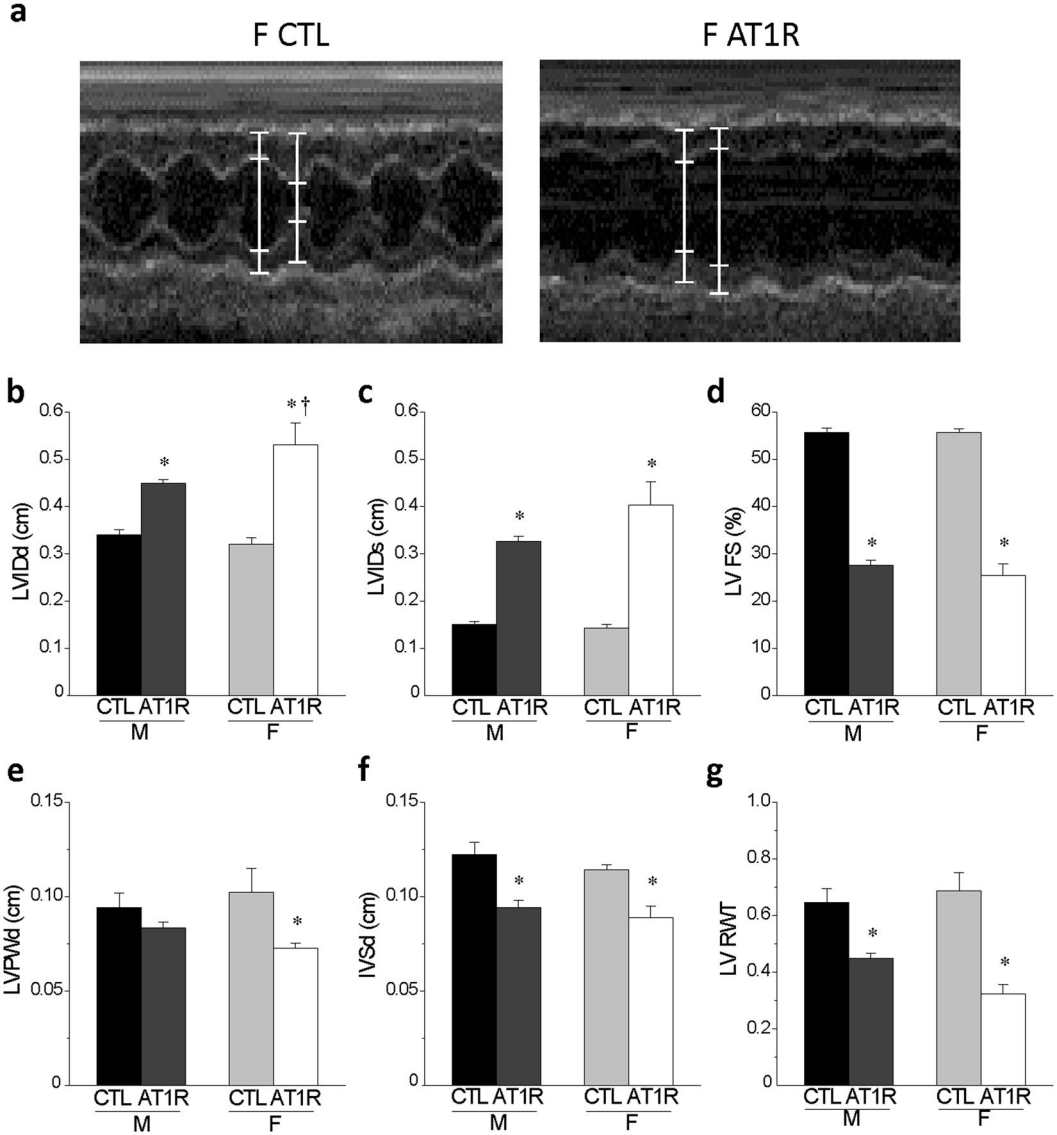
Echocardiographic data reveal that female AT1R mice develop more severe left ventricular eccentric hypertrophy and dilation than AT1R males. (**a**) Typical examples of an M-mode echocardiography recordings in female CTL and AT1R mice. (**b**,**c**) The left ventricular internal diameter at end-diastole (LVIDd) and end-systole (LVIDs) were calculated from the echocardiography data obtained from CTL and AT1R mice of both sexes. (**d**) The LV fractional shortening representing the systolic function was calculated as follows: LV FS (%) = [(LVIDd − LVIDs)/LVIDd] × 100. (**e**,**f**) The echocardiographic data were also used to assess the LV posterior wall thickness (LVPWd) (**e**) and the interventricular septum (IVSd) (**f**) for each groups. (**g**) The relative wall thickness was determined as follows: LV RWT = (IVSd + LVPWd)/LVIDd. These data indicate that AT1R stimulation leads to more pronounced eccentric hypertrophy and dilation in females than males (*p < 0.005 *vs*. sex-matched CTL, ^†^p = 0.05 *vs*. M AT1R) (M: CTL N = 7, AT1R N = 7; F: CTL N = 5, AT1R N = 8). Echocardiography values of male CTL and AT1R mice presented in this figure represent a subset of the data published in^22^.

Figure 4 summarized functional and morphological data calculated from M-mode echocardiographic measurements obtained in CTL and AT1R mice of both sexes. Left ventricular fractional shortening (LV FS, an index of systolic function) was similarly decreased (~53%) in both AT1R groups compared to CTL. However, female AT1R mice had larger increase in left ventricular internal dimension at diastole (LVIDd, F: 66% vs. M: 32%, p = 0.05, vs. M-ATR) and systole (LVIDs, F: 183% vs. M: 116%, p = 0.07, vs. M-ATR) and thinning of the left ventricular posterior wall thickness (LVPWd, by 29%, p = 0.003 vs. F-CTL) but similar decrease (~23%) in the inter-ventricular septal thickness at end diastole (IVSd) compared to AT1R males. Furthermore, the relative wall thickness (LV RWT) was decreased to a greater extent in female than male AT1R mice (reduction of 53% vs. 31%, respectively). Overall, these results suggest that although both male and female AT1R mice suffer from ventricular hypertrophy and dilation, the cardiac systolic function of females AT1R might be more severely compromised due to more pronounced left ventricular eccentric hypertrophy and dilation. Therefore, AT1R females could be more likely to rapidly progress towards a decompensated state compared to AT1R males.

### Ca^2+^ transients are altered differently between male and female AT1R mice

**Figure 5:**
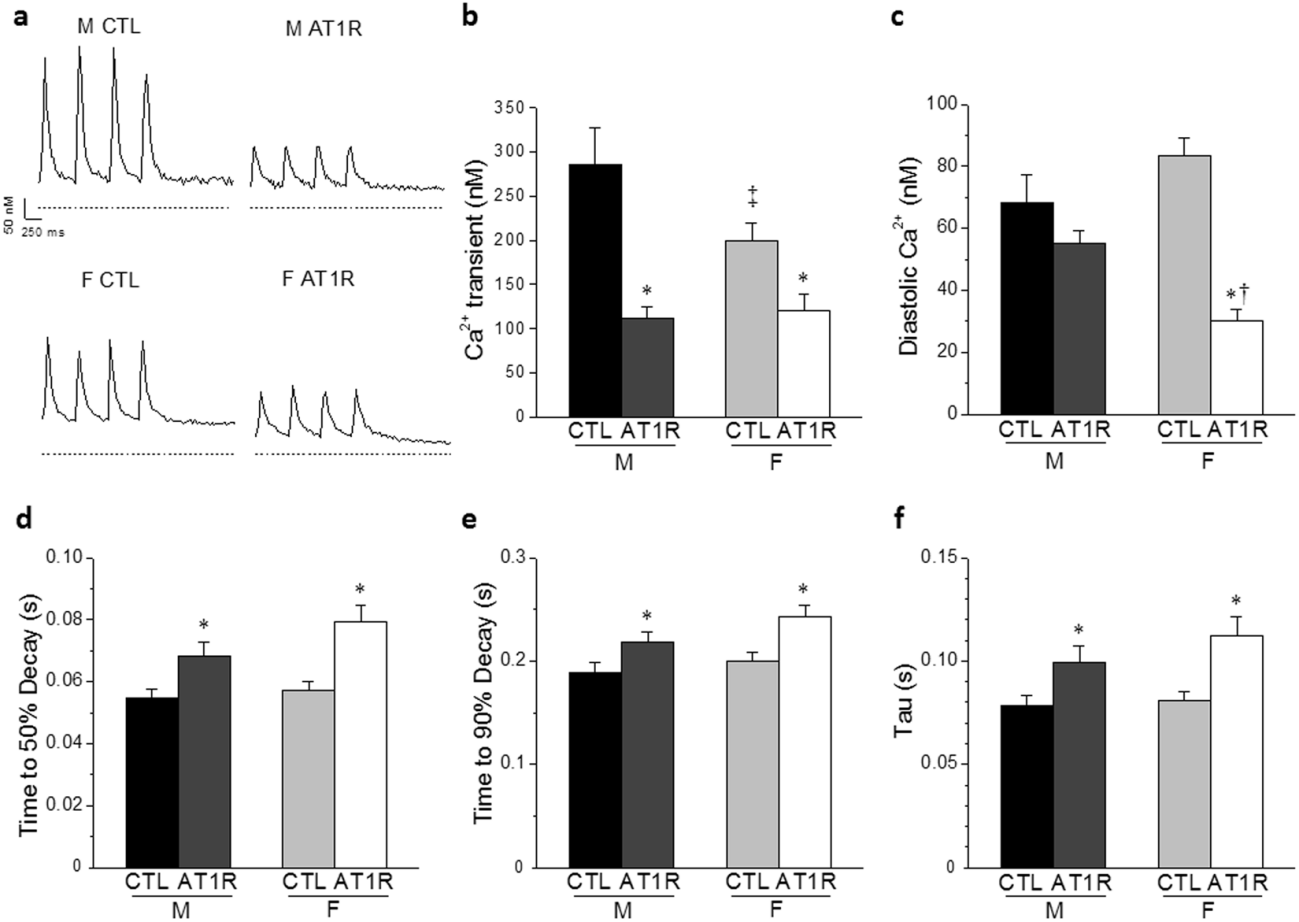
Alterations in Ca^2+^ homeostasis are greater in female than male AT1R myocytes. **(a**) Typical Ca^2+^ transients in ventricular myocytes of male and female AT1R and control mice are shown. The dashed line indicates 0 nM Ca^2+^. Ca^2+^ transients (**b**), diastolic Ca^2+^ (**c**), time to 50% decay (**d**), time to 90% decay (**e**) and tau (**f**) were measured in CTL and AT1R myocytes of both sexes (*p < 0.05 *vs*. sex-matched CTL, ^†^p = 0.005 vs. M AT1R, ^‡^p = 0.01 *vs*. M CTL) (M CTL n = 18, M AT1R n = 28, F CTL n = 28, F AT1R, n = 18).

**Figure 6:**
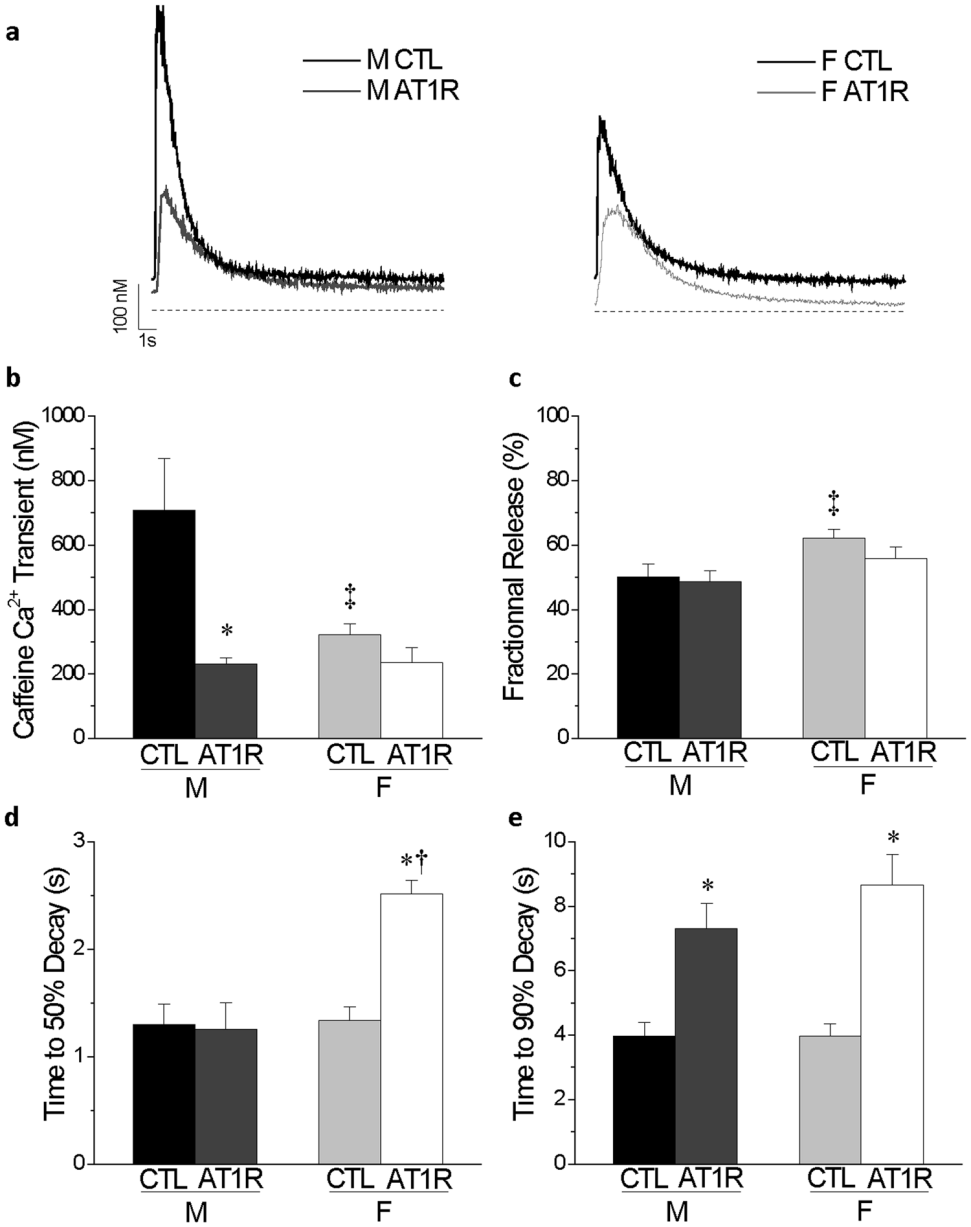
Caffeine-induced Ca^2+^ transients are differently affected in male and female AT1R myocytes. (**a**) Representative recordings of caffeine-induced Ca^2+^ transients in ventricular myocytes of male and female CTL and AT1R mice are shown. (**b**) Mean data for caffeine-induced Ca^2+^ transients show that female have lower SR load than male in CTL conditions (^‡^p = 4 × 10^−5^) and that SR load is only reduced in male AT1R myocytes (*p = 0.0004). (**c**) Fractional release defined as the ratio of Ca^2+^ transient amplitude over the caffeine-induced Ca^2+^ transient is increased in female CTL in comparison to males (^‡^p = 0.01). Time to 50% decay (**d**) and time to 90% decay (**e**) are presented (*p < 0.0005 *vs*. sex-matched CTL, ^†^p = 2 × 10^−5^ *vs*. M AT1R, ^‡^p≤0.01 *vs*. M CTL) (M CTL n = 18, M AT1R n = 22, F CTL n = 28, F AT1R n = 14).

Considering that Ca^2+^ handling is intrinsically linked to cardiomyocyte function and that AT1R female hearts show a more pronounced dilation, we sought to determine whether a poorer Ca^2+^ handling in AT1R females could be associated with their higher mortality. Our results show that in CTL myocytes, the amplitude of the Ca^2+^ transient was smaller in female mice in comparison to males (Fig. 5a,b). In male AT1R myoyctes, the amplitude of the Ca^2+^ transients was reduced in comparison to male CTL, reaching the same level of AT1R females. Furthermore, when estimating the sarcoplasmic reticulum (SR) Ca^2+^ load by application of caffeine, the amplitude of the caffeine-induced Ca^2+^ transient was dramatically reduced in AT1R male but not AT1R female cardiomyocytes, in comparison to sex-matched CTL (Fig. 6a,b). Importantly, in controls, the amplitude of the caffeine-induced Ca^2+^ transient was already considerably smaller in female CTL compared to male CTL and thus, overexpression of AT1R did not further affect the amplitude in females as it did in males. Consequently, the fractional release (Fig. 6c), the amount of Ca^2+^ released (Ca^2+^ transient) divided by the total amount of Ca^2+^ available (caffeine-induced Ca^2+^ transient), was higher in female CTL in comparison to male CTL. Altogether, these findings suggest that female controls have a much lower SR Ca^2+^ load than males and that this lower Ca^2+^ reserve was not further decreased by AT1R overexpression. Concurrently, it was also observed that diastolic Ca^2+^ concentration (Fig. 5c) was lower in female AT1R myocytes, which suggests that myofilament contraction following Ca^2+^ release could be compromised leading to a systolic dysfunction.

When evaluating cytosolic Ca^2+^ extrusion through analysis of the times to 50%, 90% decay and the Ca^2+^ transient decay (tau), we found that time to 90% decay and tau were prolonged to a similar extent in AT1R mice of both sexes in comparison to their respective controls (Figs 5e,f and 6e). However, in female AT1R myocytes, there was a strong trend (p = 0.058) for the time to 50% decay to be longer compared to AT1R males, suggesting they suffer from a greater defect in cytosolic Ca^2+^ extrusion process (Fig. 5d). This phenomenon was even more evident when measuring the time to 50% decay on the caffeine-induced Ca^2+^ transient (Fig. 6d). Indeed, only female AT1R mice showed a significantly slower time to 50% decay (p < 0.0001) supporting a poorer Ca^2+^ reuptake and extrusion mechanism in female AT1R mice.

### Spontaneous Ca^2+^ release and Ca^2+^ sparks are more frequent in female AT1R mice

**Figure 7:**
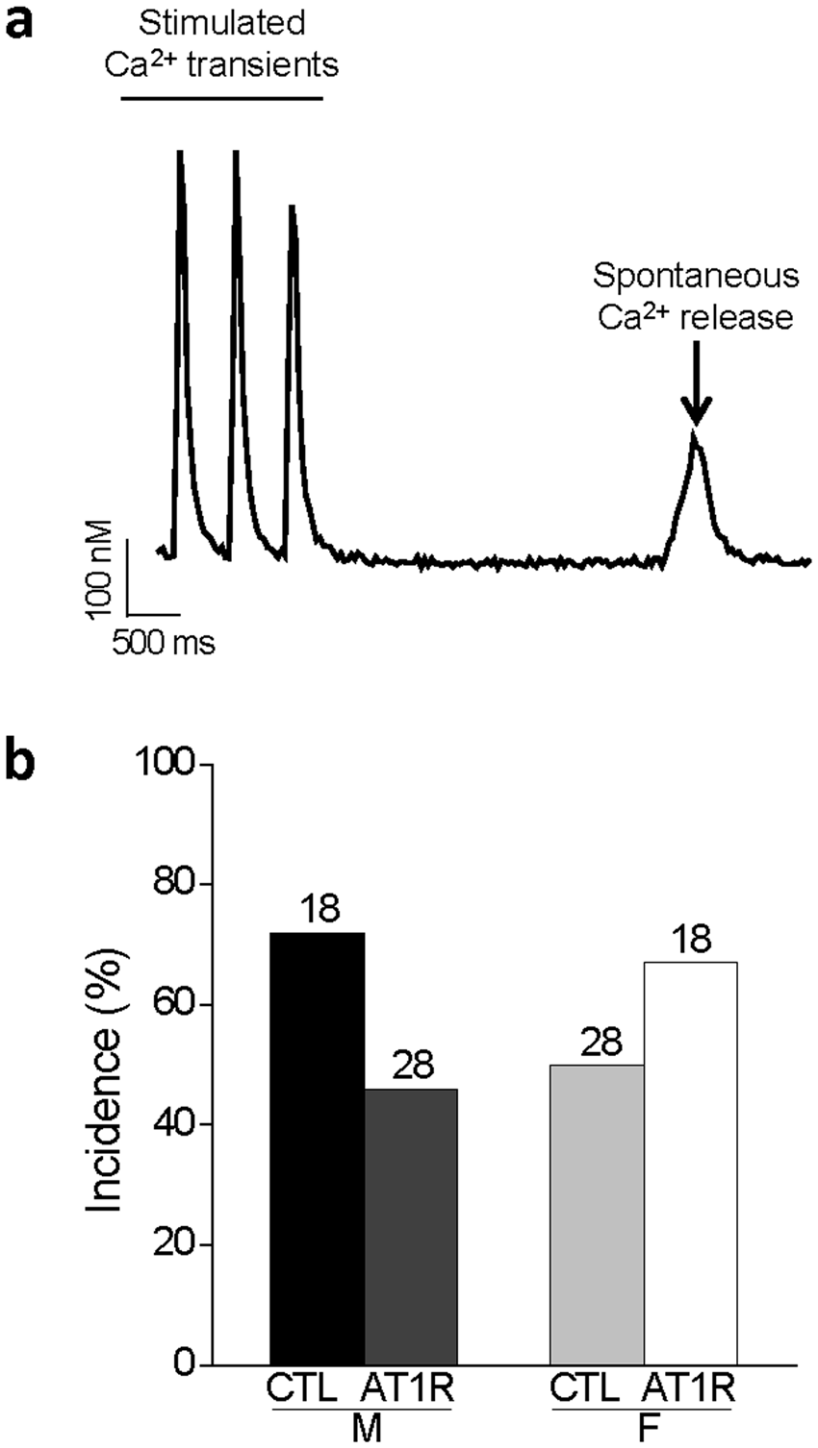
Spontaneous SR Ca^2+^ release in AT1R mice. (**a**) Typical example of a spontaneous Ca^2+^ release recorded following a stimulated Ca^2+^ transient in a female AT1R myocyte. (**b**) Bar graphs presenting the incidence of spontaneous Ca^2+^ release in myocytes from male and female CTL and AT1R mice. AT1R females have an increase in spontaneous Ca^2+^ release compared to sex-matched CTL and AT1R males although the difference was not statistical significance (p = 0.149, GLIMMIX procedure). Values above the bar graphs represent the number of cells.

During the acquisition and analysis of Ca^2+^ transient data, we observed differences in the incidence of spontaneous SR Ca^2+^ release between the groups. These events were quantified and reported in Fig. 7, which also illustrates a representative spontaneous Ca^2+^ release recorded following stimulated transients in a female AT1R myocyte. Specifically, in CTL female mice there was a smaller number of cells that display spontaneous activity compared to males. This result was expected since a smaller SR Ca^2+^ load was measured in CTL females compared to their male counterparts (Fig. 6b). However, in AT1R mice the number of cells with spontaneous Ca^2+^ activity was higher in females, although not statistically different, suggesting that in females, but not males, AT1R stimulation promotes SR Ca^2+^ leak.

**Figure 8:**
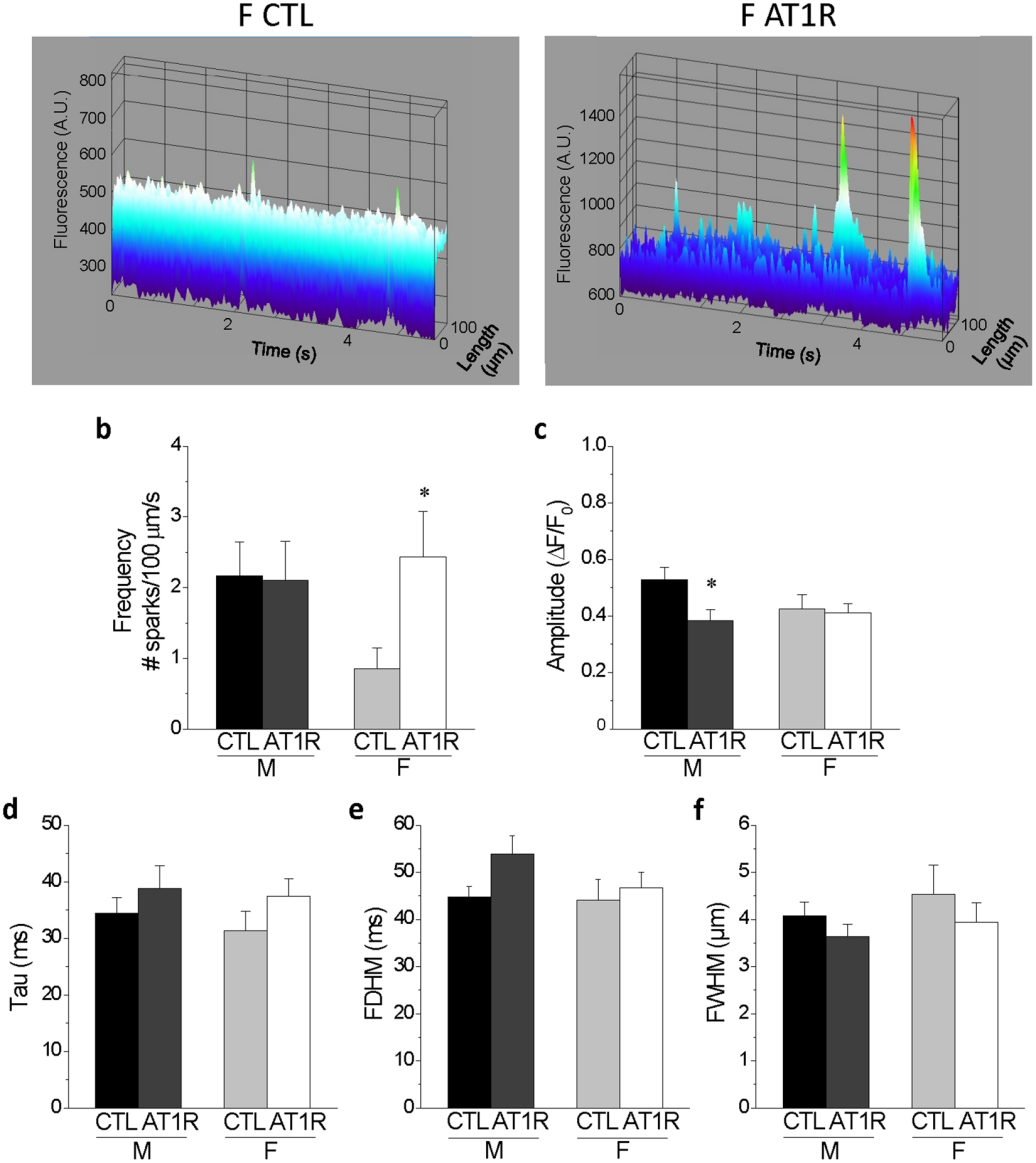
Ca^2+^ sparks frequency is increased in female AT1R myocytes in comparison to female CTL. **(a**) Representative Ca^2+^ sparks recorded in ventricular myocytes isolated from female CTL and AT1R mice. (**b**) AT1R overexpression caused no change in sparks frequency in male myocytes (CTL n = 31, AT1R n = 13) whereas the frequency significantly increased in female AT1R (n = 20) in comparison to female CTL (n = 22) (*p = 0.029). (**c**) Sparks amplitude decreased in male AT1R (n = 11) compared to CTL (n = 24) (*p = 0.025) but did not differ between females (CTL n = 12, AT1R n = 18). (**d–f)** AT1R overexpression did not affect tau (**d**), FDHM (**e**) or FWHM (**f**).

Accordingly, in order to better assess these spontaneous events, we performed rapid line-scan confocal microscopy using the Fluo-4 Ca^2+^ dye, adapted from Fares *et al*.^25^ to evaluate Ca^2+^ spark amplitude and frequency in quiescent cells of the different groups. Figure 8 shows that the spark frequency is dramatically increased in female AT1R (2.4 ± 0.6 sparks/100 µm/sec) compared to female CTL (0.85 ± 0.3 sparks/100 µm/sec, p = 0.029) mice, suggesting that there is more SR Ca^2+^ leak in AT1R females. Interestingly, in AT1R males there is a reduction in the amplitude of Ca^2+^ sparks (ΔF/F_0_: 0.38 ± 0.04) compared to CTL males (ΔF/F_0_: 0.53 ± 0.21, p = 0.025) (Fig. 8c). Along with a similar spark frequency between AT1R and CTL males, this reduction in amplitude would help lower arrhythmogenesis in AT1R males in comparison to AT1R females. Of note, the tau, the full duration at half maximum (FDHM) and the full width at half maximum (FWHM) were not affected by either AT1R overexpression or sex (Fig. 8d–f). Overall, the findings from these Ca^2+^ handling experiments show that female controls have smaller Ca^2+^ transients and SR Ca^2+^ load than male controls. In addition, in females AT1R stimulation compromises more severely the cytosolic Ca^2+^ extrusion mechanisms and promotes diastolic SR Ca^2+^ leak. Altogether, these effects could make females more susceptible to the negative impact of AT1R-induced eccentric hypertrophy and dilation.

## Discussion

ANGII plays a critical role in regulating cardiovascular function in both health and disease and is a main effector in the progression of HF. Accordingly, we sought to determine how the ANGII/AT1R cascade directly affected cardiac function in males and females and whether it could contribute to sex differences in the manifestation of HF. Using a mouse model which develops HF independently of hypertension through a cardiomyocyte-specific overexpression of the AT1R, we explored the direct role of ANGII overstimulation via AT1R on the heart of mice from both sexes. Interestingly, we observed that female AT1R mice have a lower survival rate and die at an earlier age than their male counterparts suggesting female sex can be associated with a higher susceptibility to AT1R activation. Furthermore, we found that female AT1R mice exhibit altered Ca^2+^ homeostasis as well as more ventricular hypertrophy and dilation. Specifically, no major differences in electrophysiological remodelling were found between males and females. However, we observed that under control conditions, females had a decreased Ca^2+^ transient and SR Ca^2+^ load thereby increasing their susceptibility to AT1R overexpression deleterious effects. Accordingly, we observed specifically in AT1R females a more severe eccentric hypertrophy associated with a dysfunction in Ca^2+^ reuptake/extrusion, an increased in Ca^2+^ sparks and evidence of SR Ca^2+^ leak; parameters that are associated with the development of ventricular arrhythmias. Overall, these results suggest that AT1R overexpression leads to a more pronounced phenotype in females that could contribute to their higher mortality rate.

Clinical studies have shown that women tend to have a lower incidence of SCD than men although this difference is abrogated with age^26^. In survivors of cardiac arrest, structural heart disease was studied, and the majority of men manifested coronary artery disease whereas women suffered mostly from non-ischemic disease such as dilated cardiomyopathy and ventricular heart disease^26,27^. Furthermore, ventricular fibrillation is not the only electrical disturbance that can lead to SCD. For instance, pulseless electrical activity (PEA) and asystole have also been associated with SCD^28^. It was shown that women more often display PEA, for which adequate therapy does not exist^29^. Indeed, defibrillation is useful for ventricular fibrillation to restore rhythm but is not for PEA, thus, women with implantable cardioverter defibrillator (ICD) are not as much protected as men from SCD as demonstrated in the MUSTT study^30^. Interestingly, our experimental data shows some parallels with these clinical observations. First, the presence of exacerbated dilated cardiomyopathy in females compared to males and a similar electrical remodelling support the notion that the higher mortality in female AT1R mice is not associated with alteration in ionic currents but rather from a mechanical dysfunction, poor Ca^2+^ handling and more severe ventricular hypertrophy and dilation, findings that would be compatible with a PEA.

Angiotensin-converting enzyme (ACE) inhibitors and ANGII receptor antagonist (ARA) are two important therapies used for the treatment of HF^31^. Interestingly, in one study, after treatment with losartan (an ARA) for 4.8 years, women were still suffering from more important residual eccentric hypertrophy compared to men despite a similar reduction in blood pressure^15^. Here, we observed that under AT1R overexpression both sexes display a similar reduction in fractional shortening, female mice also develop more important eccentric hypertrophy. Indeed, data showed that AT1R females had larger increase in LVIDd and LVIDs and, at the cellular level; their ventricular myocytes was significantly lengthened.

Under certain circumstances ANGII and hormonal regulation can accelerate aneurysm formation^32–36^. However, in the present study given that the overexpression is under the control of the α-myosin heavy chain (α-MHC) promoter, AT1R mediated effects are restricted to the cardiomyocytes without hypertension or vascular effect^22^, and under these conditions primary changes in aorta are not expected. In addition, no evidence of aortic enlargement or aneurysm progression was observed in male or female AT1R mice, ruling out the possibility that the presence of ascending aortic aneurysms and associated aortic insufficiency could explain the increased mortality in female AT1R mice.

In humans, sex differences in RAS modulation have mostly been associated to vascular effects with lesser examination of cardiac function. Androgen is thought to be associated with vasocontraction and estrogen with vasodilation, increased angiotensinogen and decreased renin, ACE and AT1R expression^37,38^. Consequently, the increase in blood pressure seen in postmenopausal women correlates with the loss of estrogen and of its suppressor effect on many RAS components, suggesting high RAS sensitivity in women. Consistent with this observation, female mice seem to be more sensitive to chronic ANGII/AT1R activation, which favours the development of a more severe eccentric ventricular hypertrophy converging towards dilation.

We report here that CTL males have higher SR Ca^2+^ load than females (Fig. 6b), which was associated with high incidence of sparks in males (Fig. 8b). This was expected as often a higher incidence of Ca^2+^ sparks concur with high SR Ca^2+^ content. However, both male and female AT1R mice had a high incidence of Ca^2+^ sparks although their SR Ca^2+^ stores were quite low and similar to that seen in CTL females. These data suggest that AT1R overexpression promoted spontaneous Ca^2+^ release from the SR. It is possible that AT1R stimulation enhanced the sensitivity/activity of the ryanodine receptor (RyR2) leading to increased incidence of Ca^2+^ sparks. In fact, changes in accessory proteins such as FKBP12/FKBP12.6, calmodulin and calsequestrin have all been shown to be implicated in RyR2 regulation during HF development^39–42^. Furthermore, posttranslational modifications in RyR2 phosphorylation, nitrosylation or oxidation have also been associated with an increase in RyR2 open probability in HF^39,43^. For example, PKA and CaMKII are known to phosphorylate RyR2 and to have a higher activity in HF models^39,40^. Therefore, it is possible that one or many of these aforementioned RyR2 regulatory mechanisms would be implicated in the higher RyR2 leakage in hypertrophied AT1R ventricles despite their low SR Ca^2+^ load. Altogether, the modifications in Ca^2+^ handling parameters, along with the eccentric hypertrophy and increased SR Ca^2+^ leak could result in weaker ventricular contraction and increase the risk of delayed afterdepolarization and arrhythmias.

We and others have previously shown that SERCA2a (sarco/endoplasmic reticulum Ca^2+^-ATPase) expression is significantly reduced in male mice with cardiomyocyte-specific AT1R or angiotensinogen overexpression^23,44^. Here, we found that AT1R stimulation, the time to 50% decay of the Ca^2+^ transient was further prolonged in females only. Since more than 90% of the Ca^2+^ extrusion is mediated by SERCA2a in rodents^28^, this result suggests that female AT1R mice may have more severe alteration in the function and expression of SERCA2a and/or phospholamban (PLB), a negative regulator of SERCA2a activity. Indeed, it was shown in human and animal models of HF that PLB could be hypophosphorylated and thus, exerts a higher repression of SERCA2a activity^45^. Together with an increase in Ca^2+^ sparks, these alterations would suggest that less SR Ca^2+^ is released during systole resulting in altered contraction efficiency.

In conclusion, our study shows that there are clear sex differences in the development of hypertrophy and heart failure. Indeed, both sexes of the AT1R mouse model develop HF even though females have higher mortality. We found that the weaker Ca^2+^ dynamics in female mice render them much more susceptible to the effects of a greater hypertrophy thereby, worsening their prognosis. These findings along with other reports on sex differences help raise awareness to a clinically important and often overlooked problem and highlight the need to better understand the mechanisms underlying sex differences in cardiovascular diseases.

## Methods

A detailed description of the methods can be found in the online data supplement.

### Animals

Heterozygous male and female C57BL/6 transgenic mice with a cardiomyocyte specific overexpression of *AGT1R* (AT1R mice) and their sex-matched wild-type littermates (CTL) were used at the age of 6–8 months. The generation of the AT1R mice was described previously^21^. *AGT1R* was expressed under the control of the murine α-MHC promoter, which is highly specific and directs transgene expression to the myocardium^21^. The use of this promoter rules out the involvement of peripheral effects in the cardiac phenotype studied. AT1R mice colony was followed from birth to 15 months of age and deaths were counted for the entire duration of the survival study. All animal protocols were conducted in accordance with the Canadian Council Animal Care guidelines and conformed to the Guide for the Care and Use of Laboratory Animals published by the US National Institutes of Health (NIH Publication No. 85-23, revised 1996). The Montreal Heart Institute Animal Care Committee has also approved the animal procedures of this study (reference number 2014-08-01).

### Surface ECG

As previously described, surface electrocardiogram (ECG) in lead I configuration were recorded at a rate of 2 kHz for 5 minutes in mice anaesthetized with 2% isoflurane^22^.

### Ventricular myocyte isolation

The ventricular myocyte isolation protocol was performed as previously described^46^. Briefly, each mouse was anesthetized with isoflurane, injected IP with 100 U heparine (1000 U/mL), heart was removed rapidly and hung on a modified Langendorff apparatus. Single myocytes were obtained following enzymatic digestion and dissociation of the right ventricle using Worthington collagenase type 2 (73.7 U/mL, Worthington Co. Ltd, Freehold, NJ, USA).

### Cellular electrophysiology

Recordings of Na^+^ current (I_Na_) and K^+^ currents were obtained using the voltage-clamp technique in whole-cell configuration at room temperature (20–22 °C) whereas L-type Ca^2+^ current (I_CaL_) was recorded at 37 °C. The voltage-clamp protocols, recording methods and data acquisition have already been described elsewhere^47–49^.

### Echocardiography

Short axis view two-dimensional guided M-mode echocardiography was performed in mice under conscious sedation with 15 µL/g IP of a 1:1 mixture of fentanyl (5 µg/mL) and droperidol (250 µg/mL) as previously described to determine cardiac systolic function and left ventricle morphological parameters^22,50^.

### Ca^2+^ transients

Myocytes were preincubated with 10 μmol/L Fura-2AM (Molecular Probes). Then, Ca^2+^ transients were recorded on field stimulated myocytes at a frequency of 2 Hz at 37 °C. The myocyte sarcoplasmic reticulum (SR) Ca^2+^ content was measured following fast perfusion (1 s) of 10 mmol/L caffeine on a quiescent cell. Recordings were acquired using Felix 4.1.0 (Photon Technologies International (PTI), Birmingham, NJ) and analyzed using Clampfit 10.2 (Molecular Devices, Sunnyvale, CA, USA).

### Ca^2+^ sparks

Ventricular myocytes were preincubated with 10 μmol/L Fluo-4AM and Ca^2+^ sparks on quiescent ventricular myocytes were acquired using a Zeiss LSM 710 laser scanning microscope in line scan mode. Ca^2+^ sparks parameters were measured and quantified using the ImageJ Sparkmaster plugin.

### Statistical analysis

Data were expressed as mean ± S.E.M, “N” indicate the number of mice and “n”, the number of cells. A one-way ANOVA followed by a Fisher’s post hoc test for comparison of the groups was performed using Origin 8.0 (OriginLab Corporation, MA, USA). Survival rate was analyzed using Cox regression model. For spontaneous Ca^2+^ release analysis, a multilevel mixed logit model was used (GLIMMIX procedure of SAS software version 9.4, SAS Institute Inc., Cary, NC, USA). The model was specified in terms of fixed effects for group, sex and their interaction. The random effects within the model were the intercept to take into account correlation within mouse. A p < 0.05 was considered statistically significant.

## Electronic supplementary material

Data Supplement
