# Supplementary material for: Angiotensin II Overstimulation Leads to an Increased Susceptibility to Dilated Cardiomyopathy and Higher Mortality in Female Mice
Source: Sci Rep. 2018 Jan 17;8:952. doi: 10.1038/s41598-018-19436-5 (PMC5772611; doi:10.1038/s41598-018-19436-5)
Supplement: Supplementary file 1 — Data Supplement [file 41598_2018_19436_MOESM1_ESM.pdf]

DATA SUPPLEMENT

SREP-17-35409A

Angiotensin II Overstimulation Leads to an Increased Susceptibility to Dilated Cardiomyopathy and Higher Mortality in Female Mice

Sophie Mathieu, Nabil El Khoury, Katy Rivard, Pierre Paradis, Mona Nemer, and Céline Fiset

SUPPLEMENTARY DATA

Supplementary Table 1. Comparison of ventricular *AGTR1* mRNA and plasma angiotensin II levels in control and AT1R mice of both sexes.

	**M CTL**	**M AT1R**	**F CTL**	**F AT1R**
Angiotensin II (pg/ml)	58 ± 11 (5)	79 ± 12 (6)	85 ± 16 (6)	77 ± 31 (6)
AGTR1 mRNA levels (relative expression)	1.0 ± 0.1 (4)	728 ± 129 (4)	1.1 ± 0.2 (4)	658 ± 123 (5)

"n" in parentheses indicates the number of mice.

SUPPLEMENTARY METHODS

Animals

Heterozygous male and female C57BL/6 AT1R (human angiotensin II type 1 receptor, *AGTR1*) mice aged of 6-8 months old (presence of ventricular hypertrophy) and their sex- and age-matched wild-type littermate controls (CTL) were used. AT1R mice colony was followed from birth to 15 months of age and deaths were counted for the entire duration of the survival study. Generation of the AT1R mice with cardiac specific overexpression of the human AT1R has been previously published [1]. *AGT1R* was expressed under the control of the murine α-myosin heavy chain promoter, which is highly specific and directs transgene expression to the myocardium. The use of this promoter rules out the

involvement of peripheral effects in the cardiac phenotype studied. All animal protocols were conducted in accordance with the Canadian Council Animal Care guidelines and conformed to the Guide for the Care and Use of Laboratory Animals published by the US National Institutes of Health (NIH Publication No. 85-23, revised 1996). The Montreal Heart Institute Animal Care Committee has also approved the animal procedures used in this study (reference number 2012-80-01).

Mouse ventricular myocyte isolation

Isolated mouse ventricular myocytes were obtained using previously published protocols [2,3]. Briefly, mice were administered heparin i.p. 15 minutes prior to anesthesia by isoflurane (2%) to avoid blood coagulation and were sacrificed by cervical dislocation. Then, their heart was rapidly excised and hung on a modified Langendorff apparatus and retrogradely perfused at 37°C (2 mL/min). The first solution, a HEPES-buffered Tyrode's solution containing (in mmol/L): 130 NaCl, 5.4 KCl, 1 $CaCl_2$, 1 $MgCl_2$, 0.33 Na_2HPO_4, 10 HEPES, 5.5 glucose (pH was adjusted to 7.4 with NaOH) was perfused. After, Ca^{2+} was washed out by changing to a Ca^{2+} free HEPES-buffered Tyrode's solution. Thirdly, the heart was digested for 21-29 minutes with the Ca^{2+} free HEPES-buffered Tyrode's solution in which was added (in mmol/L) 0.03 $CaCl_2$, 20 taurine and 0.1% bovine serum albumin (BSA, Sigma-Aldrich Co., St Louis, Mo, USA) and 73.7 U/ml type II collagenase (Worthington Co. Ltd, Freehold, NJ, USA). Lastly, Kraft-Brühe (KB) solution containing (in mmol/L): 100 K-glutamate, 10 K-asparate, 25 KCl, 10 KH_2PO_4, 2 $MgSO_4$, 20 taurine, 5 creatine, 0.5 EGTA, 5 HEPES, 20 glucose, 0.1% BSA (pH to 7.2 with KOH) was perfused for the last 5 minutes. During cell isolation solutions were equilibrated with 100%

0_2. For Ca^{2+} imaging experiments, EGTA was removed from the KB solution. Next, the heart was removed and the right ventricle free wall was minced into the KB solution. Isolated ventricular myocytes were obtained by trituration and conserved at 4°C in KB until used on the same day.

Surface electrocardiogram (ECG)

ECG were performed as previously described [4,5]. Mice were anaesthetized with isoflurane (2%). Mouse body temperature was maintained at 37°C using a heating pad. Platinum electrodes were positioned subcutaneously and surface ECG was acquired in lead I configuration at the rate of 2 kHz using the Biopac acquisition System MP100 (EMKA Technologies, Paris, France). The signal was amplified, filtered at 100 Hz (low-pass) and 60 KHz (notch filter). Data were analyzed using ECG auto v2.8.1.18 (EMKA Technologies, Paris, France). Duration of QRS complex, QT interval and heart rate was measured by a blinded observer from signal averaged ECG recordings (500-1000 cardiac cycles). The QT intervals were corrected (QTc) for the heart rate using the adapted Bazett's formula in mice ($QTc=QT/ (RR/100)^{1/2}$).[6,7]

Cellular electrophysiology

Voltage-clamp experiments were conducted using previously reported protocols [4,5,8–10]. Current recordings were obtained in whole-cell configuration using borosilicate glass pipettes (WPI, Sarasota, FL, USA) of maximum 3 MΩ resistance for I_{Na} recordings and were between 3-5 MΩ for I_{CaL} and total K^+ currents (I_{peak}). For I_{Na} recordings, the internal solution (in mmol/L): 132.5 CsF, 5 NaCl, 5 MgATP, 10 EGTA, 5 HEPES (pH adjusted to

7.2 with CsOH). Sodium current (I_{Na}) from mouse ventricular myocytes were measured at 22 ± 1°C with the external solution containing (in mmol/L): 132.5 CsCl, 5 NaCl, 1 $CaCl_2$, 1 $MgCl_2$, 20 HEPES, 10 glucose (pH adjusted to 7.35 with CsOH) as previously reported [5,10]. Adult ventricular mouse myocytes were held at -90 mV, I_{Na} was obtained by imposing 40 ms voltage step from -90 to +15 mV at a frequency of 0.2 Hz. I_{CaL} recondings were performed at 36 ± 1°C with the external solution containing (mmol/L): 145 TEACl, 10 CsCl, 2 $CaCl_2$, 0.5 $MgCl_2$, 5 HEPES, 5.5 glucose (pH adjusted to 7.4 with CsOH) and the internal solution used was composed of (mmol/L): 100 aspartic acid, 70 CsOH, 40 CsCl, 2 $MgCl_2$, 4 MgATP, 10 EGTA, 10 HEPES (pH adjusted to 7.2 with CsOH), currents were corrected by -10 mV to account for the liquid junction potential. Myocytes were held at -90 mV, a 50ms prepulse at -50mV was performed to inactive T-type Ca^{2+} currents and I_{CaL} was obtained by imposing a series of voltage steps from -50 mV to +40 mV, each lasting 250 ms at a frequency of 0.1 Hz.[9,11,12] Total K^+ currents were recorded at room temperature using the bath solution (in mmol/L): 130 NaCl, 5.4 KCl, 1 $CaCl_2$, 1 $MgCl_2$, 0.33 Na_2HPO_4, 10 HEPES, 5.5 glucose (pH was adjusted to 7.4 with NaOH) and the pipette solution (in mmol/L): 110 potassium aspartate, 20 KCl, 8 NaCl, 1 $MgCl_2$, 1 $CaCl_2$, 10 BAPTA, 4 K_2ATP and 10 HEPES (pH 7.2 with KOH), a junction potential of -10 mV was corrected during the analysis. Traces were elicited by a 500 ms voltage-clamp step applied in 10 mV increments from −110 to +50 mV, from a holding potential of −80 mV at a frequency rate of 0.1 Hz[9,13 4,8]. All currents protocols were performed and data acquired with Clampex 8.0 and 10.2 (Molecular Devices, Foster City, USA). Voltage-clamp recordings were low-pass filtered (2 KHz: I_{Na}; 1 KHz: I_{CaL} and I_{peak}) and digitized at (10,000

KHz). Series resistance were compensated at 75%. All currents were normalized to cell capacitance.

Echocardiography

Two-dimensional M-mode echocardiography was performed as described previously [4,13]. Briefly, the day before echocardiography, the mice were anesthetized (2 % isoflurane, 1 L/min O_2), and their anterior chest was shaved. The following day, echocardiography was performed using a Philips Sonos 5500 and a 15-MHz linear-array transducer (Agilent, Palo Alto, CA) in mice under conscious sedation with 15 µL/g i.p. of a 1:1 mixture of fentanyl (5 µg/ml) and droperidol (250 µg/ml). The mice were secured lightly in the left lateral decubitus position on a warming pad to maintain normothermia. To improve the near-field image for visualization of the interventricular septum, a standoff for the transducer tip was used. A 3- to 5-mm-thick 1% agarose pad was inserted between the chest of the mice and the transducer. The montage was sealed acoustically with prewarm acoustic coupling gel. Two-dimensional guided M-mode images were obtained from a short axis view at the papillary muscle level to determine the left ventricle (LV) structure and systolic function. The LV end-diastolic and end-systolic internal dimension (LViDd and LViDs, respectively) were measured and the LV fractional shortening (FS) was calculated as follows: FS (%) = [(LVIDd - LVIDs) / LVIDd] x 100. The interventricular septum and LV posterior wall thickness in diastole (IVSd and LVPWd) were also measured and the LV relative wall thickness (LV RWT) was determined as follows [(IVSd + LVPWd) / LViDd]. The LV mass was determined using the following equation: LV mass

= 1.055 [(IVSd + LViDd + LV PWT)^3 – (LVIDd)^3], where 1.055 is the specific gravity of the myocardium [14]. LV mass to body weight ratio was then calculated.

Ca^{2+} Transients

Experiments were performed accordingly to [9,15].Ventricular myocytes were incubated 15 minutes in the dark with 10 µmol/L Fura-2AM (Molecular Probes). Bath was previously coated with laminin and perfused at 35 ± 1°C with the HEPES-buffered Tyrode's solution containing (in mmol/L): 130 NaCl, 5.4 KCl, 1 $CaCl_2$, 1 $MgCl_2$, 0.33 Na_2HPO_4, 10 HEPES, 5.5 glucose (pH was adjusted to 7.4 with NaOH). Myocytes were field stimulated at a frequency of 2 Hz with a pair of bipolar platinum electrodes (Grass SD9 stimulator). Transients were recorded for 10 s before stopping stimulation and perfusing with a HEPES-buffered Tyrode's Ca^{2+} free solution in which was added 10 mmol/L caffeine. Caffeine induced transients were obtained by rapidly perfusing the caffeine solution for 1 s using a stylus perfusion system to estimate the SR load. Values were acquired using the 380/340 nm ratio with a fluorescence system DeltaRam (Photo Technology Int.). Rmin and Rmax values were obtained using Ca^{2+} free Tyrode solution with 5 mmol/L EGTA or 10mmol/L ionomycin with 5 mmol/L $CaCl_2$ respectively. Calibration curve assay was used to obtain the experimental Kd of 0.433 in order to calculate internal Ca^{2+} concentration. Ca^{2+} concentration were calculated using the formula: $[Ca^{2+} = K_d \left[\frac{(R-R_{\min})}{(R_{max}-R)}\right]$. Data were acquired and converted with Felix4.1.0 (Photon Technologies International (PTI), Birmingham, NJ) and analysis was executed with Clampfit 10.2 (Molecular Devices, Foster City, USA).

Ca^{2+} sparks

Rapid line-scan confocal microscopy using the Fluo-4AM Ca^{2+} dye was used to measure Ca^{2+} spark. The protocol and experimental conditions were adapted from Fares *et al.* [15]. Myocytes were incubated in the dark for 20 minutes with 10 µmol/L Fluo-4AM (Molecular Probes) and 0.05% pluronic acid. Cells were placed in the bath on the stage of a Zeiss LSM 710 laser scanning microscope in linear scan mode. Bath was perfused at 35 ± 1°C with the following solution (in mmol/L): 130 NaCl, 5.4 KCl, 1 $CaCl_2$, 1 $MgCl_2$, 0.33 Na_2HPO_4, 10 HEPES, 5.5 glucose (pH was adjusted to 7.4 with NaOH). Line scan images were acquired at 525 nm, pinhole size: 79 µm, scan speed of 264.55 lines/s, and 512 pixels/line, laser intensity of 20% using argon laser (488nm) and LSM software (version 3.2, Carl Zeiss Canada Ltd). Sparks were measured in line scan mode for ~6 s in quiescent cells. Sparks parameters (frequency, amplitude, tau, full duration at half maximum [FDHM] and full width at half maximum [FWHM]) were measured using ImageJ plugin SparkMaster. SparkMaster parameters were set speed 264.55 lines/s, 0.264 µm pixel size, background fluorescence was measured on each recording, criteria =3.8, number of intervals = 1, output = F/F_0 + sparks, extended kinetics). Extended bright lines were excluded from the analysis. A manual inspection was performed for each recording after the automated analysis to remove false positive.

REFERENCES

1. Paradis, P., Dali-Youcef, N., Paradis, F., Thibault, G. & Nemer, M. Overexpression of angiotensin II type 1 receptor in cardiomyocytes induces cardiac hypertrophy and remodeling. *Proc Natl Acad Sci* **97,** 931–936 (2000).

2. Clark, R. B. *et al.* T-tubule localization of the inward-rectifier K^+ channel in mouse ventricular myocytes: a role in K^+ accumulation. *J. Physiol.* **537,** 979–992 (2001).

3. Trépanier-Boulay, V., St-Michel, C., Tremblay, A. & Fiset, C. Gender-based differences in cardiac repolarization in mouse ventricle. *Circ. Res.* **89,** 437–444 (2001).

4. Rivard, K., Paradis, P., Nemer, M. & Fiset, C. Cardiac-specific overexpression of the human type 1 angiotensin II receptor causes delayed repolarization. *Cardiovasc Res* **78,** 53–62 (2008).

5. Grandy, S. A., Brouillette, J. & Fiset, C. Reduction of ventricular sodium current in a mouse model of HIV. *J Cardiovasc Electrophysiol* **21,** 916–922 (2010).

6. Mitchell, G. F., Jeron, A. & Koren, G. Measurement of heart rate and QT interval in the conscious mouse. *Am. J. Physiol.* **274,** H747–H751 (1998).

7. Brouillette, J., Trepanier-Boulay, V. & Fiset, C. Effect of androgen deficiency on mouse ventricular repolarization. *J. Physiol.* **546,** 403–413 (2003).

8. Brouillette, J., Clark, R. B., Giles, W. R. & Fiset, C. Functional properties of K^+ currents in adult mouse ventricular myocytes. *J. Physiol.* **559,** 777–798 (2004).

9. Rivard, K. *et al.* Overexpression of type 1 angiotensin II receptors impairs excitation-contraction coupling in the mouse heart. *Am J Physiol Heart Circ Physiol* **301,** H2018–H2027 (2011).

10. Mathieu, S. *et al.* Reduction in Na(+) current by angiotensin II is mediated by PKCα in mouse and human-induced pluripotent stem cell-derived cardiomyocytes. *Heart Rhythm* **13,** 1346–1354 (2016).

11. El Khoury, N., Mathieu, S. & Fiset, C. Interleukin-1β reduces L-type Ca^{2+} current through protein kinase Cϵ activation in mouse heart. *J. Biol. Chem.* **289,** 21896–21908 (2014).

12. Rivard, K., Trépanier-Boulay, V., Rindt, H. & Fiset, C. Electrical remodeling in a transgenic mouse model of α_{1B}-adrenergic receptor overexpression. *AJP - Heart Circ. Physiol.* **296,** H704–H718 (2009).

13. Aries, A., Paradis, P., Lefebvre, C., Schwartz, R. J. & Nemer, M. Essential role of GATA-4 in cell survival and drug-induced cardiotoxicity. *Proc Natl Acad Sci U A* **101,** 6975–6980 (2004).

14. Yang, X.-P. *et al.* Echocardiographic assessment of cardiac function in conscious and anesthetized mice. *Am. J. Physiol. - Heart Circ. Physiol.* **277,** H1967–H1974 (1999).

15. Fares, E., Parks, R. J., Macdonald, J. K., Egar, J. M. S. & Howlett, S. E. Ovariectomy enhances SR Ca^{2+} release and increases Ca^{2+} spark amplitudes in isolated ventricular myocytes. *J. Mol. Cell. Cardiol.* **52,** 32–42 (2012).
